# The effect of esketamine combined with propofol-induced general anesthesia on cerebral blood flow velocity: a randomized clinical trial

**DOI:** 10.1186/s12871-024-02446-4

**Published:** 2024-02-20

**Authors:** Shuang Yan, Qiying Li, Kaihua He

**Affiliations:** Department of Anesthesiology, The First Affiliate Hospital of Chongqing Medical University, No.1 Youyi Road, Yuzhong District Chongqing, China

**Keywords:** Esketamine, Cerebral blood flow velocity, Transcranial color-code doppler ultrasound, Induction of general anesthesia, Propofol

## Abstract

**Background:**

Esketamine is increasingly used in clinical anesthesia. The effect of esketamine on the blood flow velocity of the middle cerebral artery has a clinical guiding effect. To investigate the effect of esketamine combined with propofol-induced general anesthesia for endotracheal intubation on the blood flow velocity of middle cerebral artery and hemodynamics during the induction period.

**Methods:**

The randomized clinical trial included 80 patients aged 20-65 years who would undergo non-intracranial elective surgery under general anesthesia in our hospital from May 2022 to May 2023. The participants were divided into two groups based on anesthesia drugs: sufentanil 0.5μg/kg (group C) or 1.5mg/kg esketamine (group E). The primary outcome was variation value in average cerebral blood velocity. The secondary outcomes included cerebral blood flow velocities (CBFV), blood pressure (BP) and heart rate (HR) at four different time points: before induction of general anesthesia (T_0_), 1 min after the induction drug injected (T_1_), before endotracheal intubation (T_2_), and 1min after endotracheal intubation (T_3_). The occurrence of hypotension, hypertension, tearing and choking during induction was also documented.

**Results:**

The variation of average CBFV from time T_0_ to T_2_(ΔV_m1_) and the variation from time T_3_ to T_0_ (ΔV_m2_) were not obviously different. The median consumption of intraoperative sufentanil in group C was obviously lower than that in group E. At T_1_, the mean HR of group E was significantly higher than that of group C. At T_2_ and T_3_, the BP and HR of group E were obviously higher than that of group C. At T_2_, the CBFV in the group E were obviously higher than those in the group C. The incidence of hypotension was significantly reduced in the group E compared with the group C. There were no differences in the other outcomes.

**Conclusions:**

The induction of esketamine combined with propofol does not increase the blood flow velocity of middle cerebral artery. Esketamine is advantageous in maintaining hemodynamic stability during induction. Furthermore, the administration of esketamine did not result in an increased incidence of adverse effects.

**Trial Registration:**

15/06/2023 clinicaltrials.gov ChiCTR2300072518 https://www.chictr.org.cn/bin/project/edit?pid=176675.

## Introduction

Ketamine is an intravenous anesthetic agent that exhibits favorable sedative and analgesic properties, which has a stimulating effect on the sympathetic nerves and circulation, and ketamine is the only drug in intravenous anesthesia that can stimulate brain function. Ketamine could stimulate the sympathetic nerves, so that it causes smooth muscle relaxation and bronchiectasis. It improves lung compliance and reduces airway resistance when given intravenously. However, it may also increase bronchial secretions, which has no positive effect on asthma [1]. Ketamine has been previously reported to increase intracranial pressure (ICP), and its clinical use was limited by the end of the 20th century due to its psychiatric adverse effects [2]. Although ketamine can affect cerebral hemodynamics through direct drug effects [3], many studies have shown that ketamine use does not cause increased ICP in patients with acute brain injury, and even if a few transient increases in ICP occur, there is no evidence of harmful consequences [4]. The neurotoxicity and psychoneurological adverse effects caused by ketamine are dose- and time-dependent [5]. Ketamine combined with propofol anesthesia has been shown in studies to not result in an increase of middle cerebral artery flow velocity and there are no neurologic complications associated with the use of ketamine plus propofol anesthesia in mechanically ventilated patients [6]. At the same time, studies suggest that subanesthetic doses of ketamine may have neuroprotective effects [7, 8].

Esketamine (also known as dextroketamine) is the dextro optical isomer of ketamine. Compared to traditional racemic ketamine, its potency is higher and it has a stronger sedative and analgesic effect with fewer adverse reactions. Studies have shown that dextroketamine alone increases cerebral blood flow velocity and volume [9]. Therefore, further clinical trials are necessary to determine the effect of combining esketamine with propofol on cerebral blood flow velocity. This study aimed to investigate the effect of esketamine combined with propofol on the blood flow velocity and hemodynamics of the middle cerebral artery in patients undergoing non-neurosurgical craniotomy,and the feasibility of esketamine as an analgesic for inducing general anesthesia for endotracheal intubation, providing more evidence for the use of esketamine in neurosurgical craniotomy.

## Materials and Methods

### Study Design and Population

**Fig. 1:**
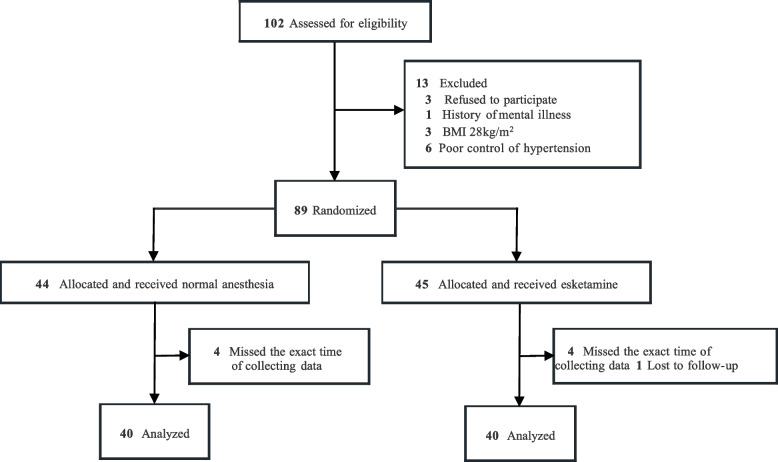
CONSORT (Inclusion procedure of the participants)

This signal-center, randomized clinical trial was conducted at the First Affiliate Hospital of Chongqing Medical University in Chongqing Province, China, from March 2022 to March 2023. The study protocol was approved by the ethics committees of the Clinical Trial Ethics Committee of the First Affiliated Hospital of Chongqing Medical University (NO.2022-072, 23/03/2022), and registered in the Chinese Clinical Trial Registratiy Center. Written informed consent was obtained from all patients or their families. This report follows the Consolidated Standards of Reporting Trials (CONSORT) reporting guideline for randomized studies. The full trial protocol is available in (Fig. 1).

### Study Procedures and Data Collection

Every patient had regular induction of intravenous anesthesia. After admission, open patient's venous access to the upper extremities. After the patient's venous access was opened, the patient's intravenous crystalloid (Ringer’s solution) was routinely given 5ml/kg. At the same time, routinely monitor heart rate (HR), oxygen saturation (SpO_2_), electrocardiogram (ECG), temperature, and monitor invasive arterial blood pressure by radial artery puncture after Allen test (+). Before induction, there had adequate nitrogen removal and oxygen then patients got slow intravenous injection of anesthetic drugs. The control group (group C) was midazolam 0.04 mg/kg, propofol 1.5 mg/kg, sufentanil 0.4 μg/kg, vicuronium bromide 0.1 mg/kg, the experimental group (group E) was midazolam 0.04 mg/kg, propofol 1.5 mg/kg, esketamine (Jiang Su Hengrui Pharmaceutical Co., Ltd., specification is 2 ml: 50 mg, Approval number: Sinopharm H20193336) 0.5 mg/kg, vecuronium bromide 0.1 mg/kg. Examination was performed using a transcranial color-code doppler (TCCD) machine (GE Venus ^TM^) fitted with 2.0-MHz sector array transducer. The ipsilateral or contralateral middle cerebral artery (MCA) was insonated through the temporal window at a depth of 46–60 mm. Observe and record the changes of middle cerebral artery blood flow velocity (V_p_, V_m_, V_d_), systolic blood pressure (SBP), average blood pressure (ABP), diastolic blood pressure (DBP) and heart rate (HR) before induction of general anesthesia (T_0_), 1 min after the induction drug injected (T_1_), before endotracheal intubation (T_2_), and 1min after endotracheal intubation (T_3_), and the incidence of adverse reactions of hypotension, hypertension, tearing and choking during the induction period. After the patient's muscles relax, the BIS index reached 40-60, the senior anesthesiologists used a visual laryngoscope (Glide Scope Ranger) to perform intubation. If BIS rises by more than 60 and mean arterial blood pressure and heart rate rise by more than 10% during intubation, sedation is performed with propofol 0.5 to 1.0 mg/kg. If systolic blood pressure is reduced by more than 20% during induction, 50 ug phenylephrine would be used each time. If HR is less than 50 times/min, atropine would be given symptomatically. After endotracheal intubation, mechanical ventilation was performed, tidal volume 6 to 8 ml/kg, respiratory rate 12 to 20 breaths/min control PetCO_2_ 35 to 45 mmHg. Anesthesia maintenance was maintained with propofol 4-10 mg/kg/h, remifentanil 15-30 μg/kg/h, sevoflurane 1%-2% inhalation, maintaining the MAC value of 0.7-1.0, adding vecuronium bromide as needed, and maintaining the BIS value at 40-55. Both groups of patients were given prophylactic antiemetic drug tropisetron 2mg intraoperatively. After the operation, all patients were sent to the PACU, and when the patient's spontaneous breathing is restored and consciousness is clear, the Steward score is greater than 6 points, the tracheal tube is removed, the oral secretion is gently sucked, and the patient is instructed to discharge sputum independently, and assist suction if necessary. Observation for at least half an hour, after the patients' vital signs are stabilized, patients are admitted to back to the ward.

### Participants

The CONSORT (https://www.goodreports.org/reporting-checklists/consort/) flowchart is shown in the Fig.1. Select patients aged 20-65 years who will undergo non-intracranial elective surgery under general anesthesia in our hospital from March 2022 to March 2023, and have an American Society of Anesthesiologists (ASA) grade I~III (I for healthy patients, II for patients with mild systemic disease, and III for patients with severe systemic disease), BMI 18-28 kg /m^2^. The types of surgery include breast and thyroid tumor resection, fracture incision reduction internal fixation and laparoscopic hysterectomy. A total of 102 participants were screened. Exclusion criteria: 1) History of psychiatric disorders and endocrine system diseases; 2) Hypersensitivity to opioids or other anesthetics; 3) History of severe heart and lung diseases; 4) Have a history of drug abuse or alcoholism; 5) `Patients at serious risk of increased blood pressure or intracranial pressure; 6) Poorly controlled or untreated hypertensive patients (arterial hypertension, resting systolic/diastolic blood pressure exceeding 180/100 mmHg); 7) Patients with untreated or undertreated hyperthyroidism; 8) Patients with craniotomy during neurosurgery.

### Randomization

Eligible participants were randomized to either esketamine or normal sufentanil (control group) using a 1:1 ratio by an online central randomization system. The randomization sequence was based on computer-generated random numbers. Patient clinical management and data collection were sequentially numbered and disclosed by health care practitioners who were not directly involved. Each code was assigned by a random number to one of the two groups: the control group or the esketamine group.

### Observation indicators

The main outcome is the variation value in average middle cerebral arterial blood flow velocity after general anesthesia induction. Define the variation of average CBFV from time T_0_ to T_2_ as ΔV_m1_, and define the variation of average CBFV from time T_3_ to T_0_ as ΔV_m2_.

The secondary outcomes include the variation value in average middle cerebral arterial blood flow velocity after endotracheal intubation and the value of V_p_, V_m_, V_d_, SBP, ABP, DBP and HR before induction of general anesthesia (T_0_), 1 min after the induction drug injected (T_1_), before endotracheal intubation (T_2_), and 1min after endotracheal intubation (T_3_), and the incidence of adverse reactions of hypotension, hypertension, tearing and choking during the induction period.

### Sample size calculation

According to the previous pre-experiment, the collected data conformed to the normal distribution, and it was concluded that the change of middle cerebral artery blood flow velocity (ΔV_m1_ =T_0_-T_2_) in the control group was 19.66±14.83 cm/s, and the experimental group was 10.83±5.99 cm/s, using G-power software, when the power was 0.90 and the significance level was 0.05, the required sample size was calculated as 34 per group. After considering potential exclusions, we opted to include 45 patients in each group. In this study, a total of 90 patients were included, and the random number table method was used to divide into 2 groups according to the order of surgery, with 45 cases in each group. A total of 40 patients were finally collected per group.

### Statistical analysis

*SPSS* 26.0 software was used for statistical analysis, measurement data were analyzed and explored, the Shapiro-Wilke test examined the variables, the mean ± standard deviation for those that conformed to the normal distribution was carried out, and the median was carried out if it did not conform to the normal distribution. Independent sample t-test was used for data that conformed to the normal distribution, and independent sample *Mann-Whitney U* test was used for data that did not conform to normal distribution. Count data are reported as numbers (%), and the χ^2^ test is used for comparison. *P*<0.05 is statistically significant.

## Results

### Baseline Characteristics and Perioperative Data

A total of 102 elective surgery patients were eligibility assessed (Fig. 1), 13 patients were excluded according to exclusion criteria, a total of 89 patients were randomly divided into two groups, 4 patients in the experimental group and 4 patients in the control group did not collect data in time during the induction period, 1 patient in the experimental group lost data during the follow-up process, and finally a total of 80 patients were randomly divided into two groups, the control group (*n*=40; median [IQR] age 48 [39-56] years) and the experimental group (*n*=40; median [IQR] age 45 [ 31-53] years) (Table 1). There were no significant differences in gender, age, weight, BMI, and duration of surgery between the two groups. The median consumption of sufentanil in the group E was obviously lower than that in the group C (45[39-50] vs25 [20-34]μg; *P*<0.001) (Table 1).

**Table 1:** General data

	**Total**	**Group C**	**Group E**	***P*-value**
Patient (%)	80(100%)	40(50%)	40(50%)	-
Gender (male: female)	80(40:40)	40(17:23)	40(22:18)	0.263
Age (years)	55(47-59)	48(39-56)	45(31-53)	0.200
Weight (kg)	63.29±9.62	63.84±9.9	62.75±9.47	0.616
Height(meters)	1.64±0.07	1.64±0.08	1.64±0.07	0.868
BMI(kg/m^2^)	23.35±2.51	23.50±2.33	23.20±2.71	0.602
Sufentanil (μg)	45(35-50)	45(39-50)	25(20-34)	<0.001
Surgery time(mins)	214(155-302)	155(120-225)	155(126-211)	0.946

*BMI* Body Mass Index (calculated as weight (kilograms) divided by height (meters) squared)

### Primary Outcome

**Fig. 2:**
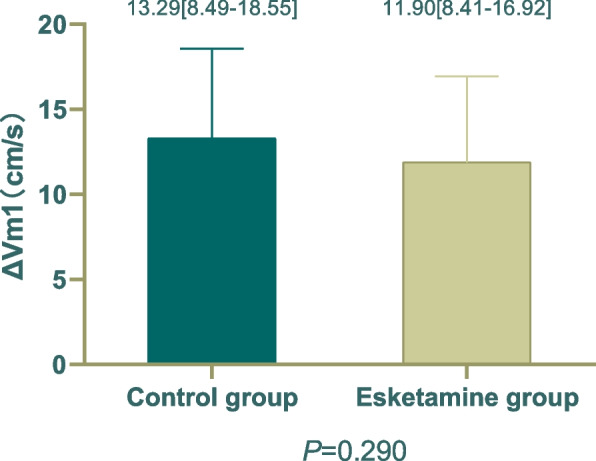
Comparison of changes in CBFV from T_0 _(Before induction) to T_2_(Before endotracheal intubation) between the two groups

**Fig. 3:**
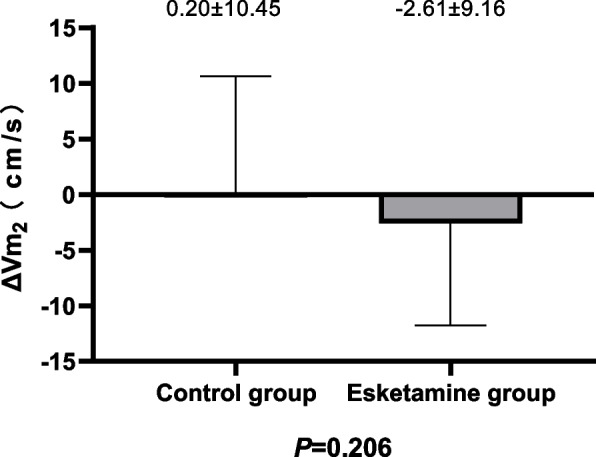
Comparison of changes in CBFV from T_3 _(1min after endotracheal intubation) to T_0_(Before induction) between the two groups

There was no significant difference in ΔV_m1_ (13.29[8.49-18.55]cm/s vs 11.90[8.41-16.92] cm/s; *P*=0.290) and ΔV_m2_ (0.20±10.45 cm/s vs -2.61±9.16 cm/s; *P*=0.206) between two groups (Figs. 2 and 3)

### Secondary Outcomes

There were no significant differences in SBP, ABP, DBP and HR at the time of T_0_ between the two groups. Additionally there were no significant differences in SBP, ABP and DBP between the two groups at the time of T_1_. At the time of T_1_, the mean HR (SD) in group E was significantly higher than that in group C (74.43±9.66 times/min vs 83.45±11.00 times/min; *P*<0.001). At the time of T_2_ and T_3_,the SBP (98[84-114]mmHg vs 115[101-131]mmHg; *P*<0.001), ABP (71.59±13.70mmHg vs85.15±14.03mmHg; *P*<0.001),DBP (58.20±11.17mmHg vs 69.18±12.01mmHg; *P*<0.001) and HR ( 67[62-79]times/min vs 84[75-89]times/min; *P*<0.001) in group E were obviously higher than that in group C (Table 2). At the times of T_0_, T_1_, T_3_, there were no significant difference in cerebral blood flow velocity (V_p_, V_m_, V_d_). At the time of T_2_, the V_p_ (50.67[42.21-61.26]cm/s vs 59.26[51.30-66.00]cm/s; *P* =0.016), V_m_ (32.10[27.46-37.86]cm/s vs 38.37 [33.93-44.27] cm/s; *P*=0.001), V_d_ (21.16[18.79-27.23] cm/s vs 28.41[22.92-32.78]cm/s;*P*<0.001) in group E were obviously higher than that in group C (Table 3).

**Table 2:** Changes in vital signs at four time points (BP [mmHg], HR [times/min])

	**Total**	**Group C**	**Group E**	***P*-value**
Before induction (T_0_)				
SBP	137(125-144)	142(128-146)	135(125-142)	0.090
ABP	96.11±8.70	97.88±9.23	94.37±7.84	0.068
DBP	76.22±8.94	77.95±9.33	74.50±8.28	0.084
HR	76.49±10.33	77.08±8.37	75.90±12.05	0.614
1 min after the induction drug injected (T_1_)				
SBP	114.09±17.75	110.83±17.30	117.35±17.82	0.101
ABP	82.74±13.18	79.96±13.15	85.52±12.77	0.059
DBP	67.06±11.67	64.53±11.80	69.60±11.11	0.051
HR	78.94±11.25	74.43±9.66	83.45±11.00	<0.001
Before endotracheal intubation (T_2_)				
SBP	107(95-120)	98(84-114)	115(101-131)	<0.001
ABP	78.37±15.37	71.59±13.70	85.15±14.03	<0.001
DBP	63.69±12.78	58.20±11.17	69.18±12.01	<0.001
HR	76(65-85)	67(62-79)	84(75-89)	<0.001
1min after endotracheal intubation (T_3_)				
SBP	142(130-154)	133(115-146)	149(140-161)	<0.001
ABP	103(93-113)	99(84-105)	108(101-117)	<0.001
DBP	85(73-93)	79(68-86)	91(79-96)	<0.001
HR	89.19±14.20	81.95±12.75	96.43±11.76	<0.001

*SBP* Systolic blood pressure, *ABP* Average blood pressure, *DBP* Diastolic blood pressure, *HR* Heart rate

**Table 3:** Changes in cerebral blood flow velocity at four time points (cm/s)

	**Total**	**Group C**	**Group E**	***P*-value**
Before induction (T_0_)				
V_p_	73.75(61.56-86.35)	69.07(59.24-83.36)	80.27(65.32-88.70)	0.057
V_m_	47.78(42.36-54.83)	45.89(38.58-53.37)	51.06(43.91-55.48)	0.072
V_d_	35.66(31.37-40.33)	34.35(28.91-39.33)	36.36(33.08-40.52)	0.132
The induction drug injected for 1 min (T_1_)				
V_p_	60.08±13.02	57.62±12.53	62.53±13.19	0.092
V_m_	38.07(31.73-41.86)	36.75(30.68-41.18)	39.53(35.59-42.88)	0.110
V_d_	27.48(21.94-31.11)	26.29(21.43-31.11)	28.01(24.27-31.20)	0.389
Before endotracheal intubation (T_2_)				
V_p_	57.46(46.58-64.53)	50.67(42.21-61.26)	59.26(51.30-66.00)	0.016
V_m_	35.47(29.54-41.54)	32.10(27.46-37.86)	38.37(33.93-44.27)	0.001
V_d_	24.95(20.58-39.60)	21.16(18.79-27.23)	28.41(22.92-32.78)	<0.001
After 1 min of endotracheal intubation (T_3_)				
V_p_	70.45(59.08-81.56)	69.00(58.83-84.66)	71.51(60.07-79.96)	0.784
V_m_	45.93(41.45-55.18)	45.86(41.29-58.03)	45.93(41.85-54.68)	0.920
V_d_	34.48(30.18-43.33)	34.48(30.59-43.69)	34.52(28.94-42.58)	0.751

*V*_p_ systolic middle cerebral artery blood flow velocity, *V*_m_ average middle cerebral artery blood flow velocity, *V*_d_ diastolic middle cerebral artery blood flow velocity

### Adverse reactions

There were no significant abnormalities in the incidence of hypertension, tearing and choking in the two groups. The incidence of hypotension in group E was significantly reduced compared to group C (11 [13.75%] vs 0 [0%];* P*<0.001) (Table 4)

**Table 4:** Adverse reactions

	**Total**	**Group C**	**Group E**	***P*-value**
Hypertension (%)	24(30%)	8(20%)	16(40%)	0.087
Hypotension (%)	11(13.75%)	11(13.75%)	0(0%)	<0.001
Tears (%)	6(7.5%)	1(2.5%)	5(12.5%)	0.201
Choking (%)	3(3.75%)	3(3.75%)	0(0%)	0.241

## Discussion

The main findings of the trial are as follows. Firstly, there was no significant difference on the mean blood flow velocity of middle cerebral artery after administration between the esketamine group and the conventional induction group, and the mean blood flow velocity of the middle cerebral artery in both groups exhibited a reduction prior to endotracheal intubation as compared to pre-induction values, indicating that esketamine combined with propofol induction had no significant advantage over conventional sufentanil combined with propofol induction in maintaining the stability of the average blood flow velocity in the middle cerebral artery. Moreover, the esketamine group did not exhibit an increase in mean blood flow velocity of the middle cerebral artery following endotracheal intubation when compared to the conventional induction group, indicating that esketamine combined with propofol anesthesia did not increase the middle cerebral artery blood flow velocity. Secondly, the results showed that the cerebral blood flow velocity in the esketamine group before endotracheal intubation was significantly higher than that in the control group, while there was no significant difference in ΔV_m1_, which may be related to the difference in the cerebral blood flow velocity before induction. Although there was no significant difference in cerebral blood flow velocity between the two groups of induction, the study size was small, and the difference in cerebral blood velocity before induction did not necessarily result in a clinically significant difference in cerebral blood velocity between the two groups prior to endotracheal intubation, which deserves further study. Furthermore, the esketamine group had a significant advantage over the conventional induction group in maintaining hemodynamics during the induction process.

General anesthesia is most commonly induced by sedatives, analgesics, and neuromuscular blockers. However, during induction of general anesthesia, the administration of sedatives often causes hypotension, particularly when potent sedatives such as propofol are utilized [10]. Tracheal intubation is an important part of general anesthesia, and opioid-combined propofol is widely used in clinical practice for anesthesia induction to avoid severe stress reactions such as sharp increase in blood pressure and abnormal rapid heart rate [11]. Therefore, the selection of appropriate anesthetic agents to ensure hemodynamic stability during induction is a crucial factor in ensuring surgical safety. At the same time, cerebral blood flow is clearly correlated with peripheral hemodynamics. Cerebral blood flow is related to the cerebral auto-regulation curve, and cerebral blood flow remains relatively stable when the mean arterial pressure is maintained at 50-160mmHg. It was generally believed that hemodynamic stability can maintain relatively stable cerebral blood flow under the automatic regulation mechanism of the brain. Studies have shown that [12] the regulatory mechanisms for maintaining CBF can be broadly divided into brain self-regulation (CA), neurovascular coupling (NVC), and vasomotor responsiveness (VMR). Changes in cerebral blood flow during anesthesia are attributed to the direct vasodilating effect of anesthetics and the partial pressure of carbon dioxide at end-expiration during mechanical ventilation. Cerebral blood flow is pressure passive beyond the autoregulatory range, and there is a risk of ischemic injury to the brain in hypotension, and cerebral edema and disruption of the blood-brain barrier in hypertension [13]. TCCD is considered a valuable tool for investigating cerebral perfusion by measuring the flow velocity of the middle cerebral artery, which plays a crucial role in distributing cerebral blood flow. A significant decrease in middle cerebral artery blood flow can serve as an indicator of overall reduction in cerebral blood flow [14]. Therefore, TCCD was used to monitor the middle cerebral artery blood flow velocity to investigate the effect of esketamine combined with propofol tracheal intubation induction on cerebral blood flow velocity.

Ketamine is an intravenous anesthetic that exhibits favorable sedative and analgesic properties, while also exerting a stimulatory effect on the sympathetic nervous system and circulation. The primary analgesic effect of ketamine is a noncompetitive antagonist to N-methyl-d-aspartate (NMDA) receptor and μ-opioid receptor [15, 16]. It does not inhibit respiratory or myocardial function or hemodynamics [17], so it is more commonly used for induction of anesthesia in special populations. Unlike the increased CBF observed with ketamine monotherapy or S-ketamine anesthesia [9, 18, 19], recent studies have shown that ketamine does not increase intracranial pressure when combined with common anesthetic drugs [20], while further studies suggest that elevated CBF occurs with ketamine alone is caused by direct vasodilation of medium cerebral vessels rather than through secondary effects due to changes in arterial carbon dioxide and/or mean arterial blood pressure [3, 21].

Esketamine, a racemic isomer of ketamine, exhibits fewer adverse effects and shorter sedation periods compared with racemic ketamine [22], for instance produces less psychiatric adverse reactions [23], while demonstrating approximately twice the efficacy of its counterpart [24]. Therefore, the clinical application of esketamine is gradually increasing, and the impact of this on cerebral blood flow remains unclear and warrants further investigation. In this study, we found that the induction of esketamine combined with propofol anesthesia has no significant advantage in maintaining stable cerebral blood flow velocity and does not increase cerebral blood flow velocity during endotracheal intubation, which may be related to the direct relaxation of cerebral blood vessels by propofol [20, 25]. Numerous studies have shown that propofol reduces cerebral glucose metabolism, cerebral oxygen metabolism, and cerebral blood flow [26, 27]. In this study, the negative impact of propofol on cerebral blood flow velocity outweighed the positive effect of esketamine, possibly due to differences in dosage administration for both drugs. The effect of different doses of esketamine combined with propofol anesthesia induction on cerebral blood flow velocity can be further studied.

The primary mechanism by which esketamine maintains hemodynamic stability is through excitation of the sympathetic nervous system, resulting in increased heart rate, blood pressure, and cardiac output. However, peripheral circulatory resistance remains largely unchanged, effectively preserving hemodynamic stability. The use of esketamine during induction of anesthesia increases the perfusion index and mean arterial pressure after induction [28]. In this study, it was also concluded that esketamine combined with propofol induced general anesthesia for endotracheal intubation could maintain hemodynamic stability, and did not increase the incidence of adverse reactions compared with conventional anesthesia induction. This conclusion is the same as most findings [28, 29].

The study had some limitations. Firstly, the trial was conducted in a single centre and further multicentre studies are needed. Secondly, the sample size of the study was small, and there was a certain difference in the basic value of the randomization group, resulting in the loss of clinical significance of some of the results. In addition, biological samples such as blood were not taken, which may help determine the possible mechanisms behind esketamine. Fourth, the main indicator of the study is only the cerebral blood flow rate, and the effect of esketamine on intracranial pressure, cerebral oxygen metabolism, and cerebral glucose metabolism needs to be further studied. Finally, in terms of the opposite effects of esketamine and propofol on cerebral blood flow, further titrations of the dose of the two need to be explored to find a more appropriate dosage.

## Conclusion

This randomized controlled trial showed that esketamine combined with propofol for induction of endotracheal intubation general anesthesia does not have significant advantages in maintaining cerebral blood flow velocity, and does not cause an increase in cerebral blood flow velocity, which can be safely used for endotracheal intubation anesthesia induction and provides more evidence for the further application of esketamine in neurosurgery. Moreover, esketamine does maintain hemodynamic stability during induction. Further studies with larger sample sizes are needed to confirm the effect of eketamine on the rate of blood flow to the brain.

## Data Availability

The data that support the findings of this study are available from the corresponding author upon reasonable request.

## Abbreviations

CBFV: Cerebral blood flow velocities
BP: Blood pressure
HR: Heart rate
ICP: Intracranial pressure
SpO_2_: Oxygen saturation
ECG: Electrocardiogram
MCA: Middle cerebral artery
V_p_: Systolic middle cerebral artery blood flow velocity
V_m_: Average middle cerebral artery blood flow velocity
V_d_: Diastolic middle cerebral artery blood flow velocity
SBP: Systolic blood pressure
ABP: Average blood pressure
DBP: Diastolic blood pressure
BIS: Bispectral
MAC: Minimum alveolar concentration
PACU: Post-anesthesia care unit
ASA: American society of anesthesiologists
BMI: Body mass index
CA: Brain self-regulation
NVC: Neurovascular coupling
VMR: Vasomotor responsiveness
NMDA: N-methyl-d-aspartate
